# Development of TiO_2_–CaCO_3_ Based Composites as an Affordable Building Material for the Photocatalytic Abatement of Hazardous NO_x_ from the Environment

**DOI:** 10.3390/nano14020136

**Published:** 2024-01-06

**Authors:** Madhan Kuppusamy, Sun-Woo Kim, Kwang-Pill Lee, Young Jin Jo, Wha-Jung Kim

**Affiliations:** 1 GOONWORLD Corporate Research Institute, Dong-gu, Daegu 41065, Republic of Korea; mitmadhan@gmail.com (M.K.); kplee@knu.ac.kr (K.-P.L.); 2 Department of Chemistry Education, Chosun University, Gwangju 61452, Republic of Korea; swookim@chosun.ac.kr; 3 Korea Conformity Laboratories, Daegu 42994, Republic of Korea; yjjo4u@kcl.re.kr

**Keywords:** photocatalysts, NO_x_, urban environment, depollution, construction materials, composite

## Abstract

This study explores the depollution activity of a photocatalytic cementitious composite comprising various compositions of n-TiO_2_ and CaCO_3_. The photocatalytic activity of the CaCO_3_–TiO_2_ composite material is assessed for the aqueous photodegradation efficiency of MB dye solution and NO_x_ under UV light exposure. The catalyst CaCO_3_–TiO_2_ exhibits the importance of an optimal balance between CaCO_3_ and n-TiO_2_ for the highest NO_x_ removal of 60% and MB dye removal of 74.6%. The observed trends in the photodegradation of NO_x_ removal efficiencies suggest a complex interplay between CaCO_3_ and TiO_2_ content in the CaCO_3_–n-TiO_2_ composite catalysts. This pollutant removal efficiency is attributed to the synergistic effect between CaCO_3_ and n-TiO_2,_ where a higher percentage of n-TiO_2_ appeared to enhance the photocatalytic activity. It is recommended that CaCO_3_–TiO_2_ photocatalysts are effectiveness in water and air purification, as well as for being cost-effective construction materials.

## 1. Introduction

A flurry of research activities is directed at the removal of nitrogen oxides (NO_x_), one of the main pollutants in the atmosphere, due to their toxic effects on health and a series of other ecological and environmental problems [1]. Various methodologies have been developed, such as selective non-catalytic reduction, selective catalytic reduction, scrubbing, and adsorption, to control NO_x_ emissions [2]. However, the above-mentioned technologies for NO_x_ degradation are costly and cause secondary pollution [3]. One of the promising techniques for enhancing the photocatalytic performance of heterojunction semiconductor catalysis has been attempted to resolve these issues [4,5,6]. In the process of development to real applications, titanium dioxide (TiO_2_) photocatalysts have also been included in concrete engineering toward degradation of NO_x_ in the atmosphere [7]. Despite a few advancements in the production of commercial photocatalytic types of cement with the inclusion of nano titanium dioxide (n-TiO_2_) particles, the utility of photocatalytic cement/concrete is limited, especially in terms of its efficiency in dealing with air pollution and cost-effectiveness [8,9]. Many reports revealed the efficacy of TiO_2_ photocatalysts in cementitious materials (with TiO_2_ loadings from 1 to 10 wt.% as a fraction of the cement content) [10]. These TiO_2_ preparations of photocatalytic cement/concrete materials have been proven to be efficient in depolluting the environment by degrading NO_x_ (de-NO_x_ effect). The successful application of n-TiO_2_ cement-based materials in engineering has been well demonstrated. The pavement prepared with n-TiO_2_ cement-based materials in China exhibited a good ability to remove NO_x_ exhaust from vehicles [11]. The road exhaust index of nano-TiO_2_ concrete pavement materials in Milan, Italy, was determined, and it was found that a reduction of 60–70% in the pollution index has been witnessed [12]. A cricket stadium in Dubai sports city is coated with nano-TiO_2_ white cement in buildings to provide improved air quality [13]. However, there are a few key questions regarding maximizing the photocatalytic efficiencies and cost-effectiveness of n-TiO_2_-incorporated photocatalytic cement/concrete in real environmental conditions.

The n-TiO_2_ has several advantages and features, like stable physical and chemical properties, non-toxic nature, low cost, and adequate photocatalytic activities under ultraviolet light [14,15,16,17,18]. n-TiO_2_ can be easily agglomerated when it is mixed with cementitious materials [19]. The highly alkaline and calcium-rich cement environment promotes TiO_2_ agglomeration, decreases the catalyst surface area, and causes the surface precipitation of calcium hydroxide/calcium carbonate [20]. As a consequence of poor dispersibility and/or occlusion of TiO_2_ by cement hydrates, high loadings of n-TiO_2_ are required to achieve high photocatalytic efficiency, which leads to higher costs. These aspects make the effective dispersion of TiO_2_ in cement/concrete a challenging task [21]. It should also be kept in mind that n-TiO_2_ has a poor response under visible light, which limits the development of photocatalytic cement-based materials in natural light scenarios [22]. We envisage that there needs to be a good strategy for balancing high photocatalytic efficiency and cost-effectiveness in real-world conditions.

The aim of this study is to develop an efficient and cost-effective photocatalytic cement material along with TiO_2_ as a promotor and immobilize the resultant composite onto the surface of a pre-formed mortar (which can absorb the composite particles on its surface). Calcium carbonate (CaCO_3_) is a kind of inorganic, nonmetallic mineral (mostly as calcite) material with abundant reserves and also derived from biowaste chicken eggshells; calcite materials have advantageous characteristics, such as high whiteness and low cost, and are considered superior to most non-metallic minerals in cost-effective performance [23,24]. The development of CaCO_3_–nano-TiO_2_ composite photocatalysts using CaCO_3_ as the additive to TiO_2_ has been demonstrated to reduce the agglomeration of TiO_2_ particles, improve the recyclability and reusability performance of nano-TiO_2_, and enhance the photocatalytic efficiency of nano-TiO_2_ particles. In the literature, a few techniques, such as hydrolytic deposition, sol-gel, and chemical precipitation methods, have been reported for the preparation of TiO_2_–n-CaCO_3_. The hydrolysis of TiCl_4_ on the surface of CaCO_3_ and the resultant composite exhibited the maximum photocatalytic degradation efficiency (95%) of Rhodamine B (10 ppm) upon UV irradiation [21,25,26]. The preparation of CaCO_3_–TiO_2_ composite particles has been demonstrated through carbonation in a TiO_2_ system [27]. The preparation of CaCO_3_–TiO_2_ composite particles with hydrophobic agglomeration has been detailed [28,29]. The CaCO_3_–TiO_2_ composite was prepared using the chemical precipitation method by coating the surface of CaCO_3_ with TiO_2_ using titanium sulfate as the titanium source [30]. The resulting product, CaCO_3_–TiO_2_, has been proven to have high ultraviolet absorption capacity. However, the above methods for the preparation of CaCO_3_–TiO_2_ are complex, time-consuming, and costly. It should be mentioned that the utility of CaCO_3_–TiO_2_ composites for construction material development is scarce.

The photocatalytic CaCO_3_–TiO_2_ composites were optimized to achieve high catalytic efficiency for NO_x_ degradation, and aqueous methylene blue (MB) photodegradation efficiencies were hardly reported. In this work, we develop a CaCO_3_–TiO_2_ composite with various weight percentages (wt.%) of catalyst-based cement mortar and surface coating on the mortar to enhance the efficiency and also the production of large-scale, cost-effective construction materials.

## 2. Materials and Methods

### 2.1. Materials

Anatase TiO_2_ (COTIOX KA-100, Cosmo Chemical, Ulsan, Republic of Korea), CaCO_3_ (Taekyung BK, Seoul, Republic of Korea), and Methylene Blue (Samchun, Pyeongtaek, Republic of Korea) were used without further process.

### 2.2. Preparation of CaCO_3_–n-TiO_2_ (CT) Catalysts

To prepare the CaCO_3_–n-TiO_2_ composite, various weight percentages (wt.%) of CaCO_3_ (C) and TiO_2_ (T), such as CT-1(C 70-T 30), CT-2 (C 60-T 40), CT-3 (C50-T 50), CT-4 (C 40-T 60), and CT-5 (C 30-T 70), were mixed using a mortar and pestle for 30 min at room temperature. 

### 2.3. Preparation of Photocatalyst Included Mortar

Mortar specimens were prepared following the compressive strength test method (KS L 5105) of hydraulic cement mortar. In a typical preparation, cement and standard sand were used in a weight ratio of 1:2.45, and the amount of water was 60% based on the weight percentage of the cement. The mortar specimen size was 100 mm long, 50 mm wide, and 5 mm high. The photocatalyst was applied on the surface of the early-aged mortar (cured in water for 7 days and dried in the air for 2 days) thin plate samples; toward that, approximately 0.5 g of the CT composite photocatalyst was dispersed in 1 g of water and directly applied uniformly on the mortar surface as a coating and dried at 80 °C. For testing the mortars mixed with photocatalysts, in addition, the calculated amount of photocatalyst was mixed with 5, 10, and 15% based on the weight percentage of cement. For the NO_x_ reduction test (KS L ISO 22197-1), water curing at 25 °C was performed for 7 days. The fabricated cement mortar specimen is shown in Figure S1.

## 3. Characterization

The phase purity of the composite samples was confirmed using room temperature XRD (Panalytical, United Kingdom) measured with Cu–Kα (λ = 1.5418 Å) radiation and a 0.02° scan step size from (2θ) 10 to 80°. Field emission scanning electron microscopy (FE-SEM) was analyzed with Hitachi and X-ray photoelectron spectroscopy (XPS), Thermo Fisher, Waltham, MA, USA (NEXSA). The optical properties were analyzed with a UV–Vis spectrophotometer S-3100 (Scinco Co., Ltd., Seoul, Republic of Korea). The photo-catalytic performance of the photocatalysts was investigated by irradiating the MB solution under a UV lamp (20 W, λ = 352 nm) purchased from SANKYO DENKI CO., Ltd., Tokyo, Japan. The concentration of MB was assessed with a UV–Vis spectrophotometer S-3100 (Scinco Co., Ltd.) at 665 nm. The concentrations of NO_x_ were recorded with the chemiluminescence technology of the NO_x_ analyzer—Serinus 40.

### 3.1. Photodegradation of MB

The experiments were performed following the specifications of KS L ISO 10678. Before irradiation, 0.5 g of photocatalyst powder was added to the MB solution prepared with a concentration of 10 mg/L. The bulk MB (200 mL) was prepared in water, and the TiO_2_ sample was dispersed with continuous stirring for 10 min. The dispersion was kept in a dark chamber for 1 h to ensure adsorption–desorption equilibrium. Subsequently, the mixed solution was irradiated under UV light and stirred constantly, while the temperature inside the stainless chamber was maintained at 25 °C. At periodic time intervals, a catalyst solution was taken successively from the mixture using an appropriate filter mounted on a syringe to separate photocatalyst particles. The concentration of MB was assessed by recording the absorbance of the solution at 665 nm.

### 3.2. Photodegradation of NO_x_

NO_x_ removal experiments were carried out according to the KS L ISO 22197-1 standard. The photoactivity of the prepared samples was measured through a photoreactor placed in a stainless box whose dimensions were 620 mm × 430 mm × 285 mm. The photoreactor was 430 mm long, 100 mm wide, and 40 mm high. On the top cover of the stainless box, light sources were installed to induce photoactivity. The size of the sample in the photoreactor was 100 mm long, 50 mm wide, and 5 mm high. The distance from the top surface of the mortar thin plate sample to the optical window of the photoreactor was approximately 10 mm. The prepared mortar sample was introduced into the photoreactor, and then the mass flow controller was used to adjust the flow rate of NO gas, water vapor, and air. The concentrations of NO_x_ were recorded with the chemiluminescence technology of the NO_x_ analyzer—Serinus 40. The photocatalytic experiment was carried out when NO_x_ was stabilized at 1000 ppb for 30 min after reaching the adsorption–desorption equilibrium in dark conditions. A UV lamp emitting UV rays between 310 nm and 400 nm was used to illuminate the photocatalytic mortar sample surface at an intensity of 1000 µW/cm^2^ for 6 h. Afterward, the UV light was turned off, and the NO_x_ concentration went back to the stabilized concentration level monitored for 30 min.

### 3.3. Possible Photocatalytic Mechanism of MB

**Figure 1:**
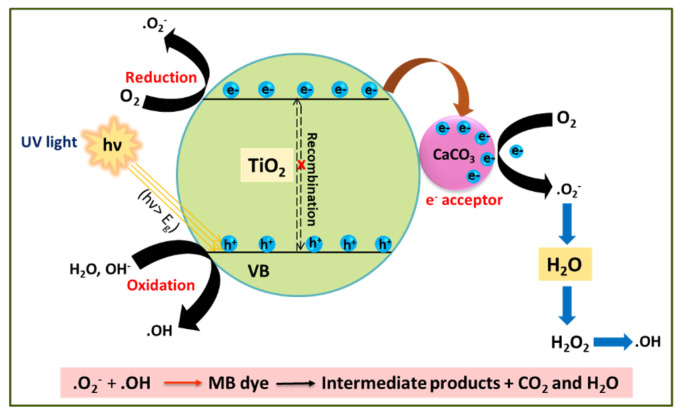
MB degradation possible mechanism of TiO_2_–CaCO_3_ composite.

The photocatalytic mechanism of MB dye degradation by the TiO_2_–CaCO_3_ composite is depicted in Figure 1.

The TiO_2_ component of TiO_2_–CaCO_3_ acts as a photocatalyst to mainly perform the photocatalytic function. Under the influence of UV light irradiation, the high-energy photons activate to create photoinduced electrons (e^−^) and holes (h^+^) in TiO_2_. The negatively charged electrons are energized to cross the band gap (Eg) and jump into the CB, leaving behind positively charged holes in the VB. In the presence of CaCO_3_, the CB electrons migrate towards the surface of CaCO_3_ (CaCO_3_ acts as an e-sink). This electron transfer process can prevent the e^−^-h^+^ pair recombination. Then, the electrons trapped in CaCO_3_ can react with adsorbed O_2_ to generate free superoxide radicals (●O_2_^−^). Simultaneously, photogenerated holes combine with H_2_O or the adsorbed hydroxyl ion (OH^−^) molecules to form hydroxyl radicals (●OH). Finally, the generated free radicals (●OH, ●O_2_^−^, h^+^) with high oxidizing ability would destroy the MB molecules into low-weight intermediates. These smaller compounds are further degraded to similar molecules, such as CO_2_ and H_2_O.

The entire photocatalytic process for MB degradation is summarized in the following equations:

TiO_2_+ hν (UV) → TiO_2_ (e^−^_CB_ + h^+^_VB_) (1)

TiO_2_ (e^−^_CB_ + h^+^_VB_) + CaCO_3_ → TiO_2_ (h^+^_VB_) + CaCO_3_ (e^−^) (2)

CaCO_3_ (e^−^) + O_2_ → CaCO_3_ + ·O_2_^−^ (3)

TiO_2_ (h^+^_VB_) + OH^−^→ TiO_2_ + ·OH (4)

O_2_·^−^ + H^+^ → ·OOH (5)

2·OOH → O_2_ + H_2_O_2_ (6)

H_2_O_2_ + O_2_^−^ → ·OH + OH^−^ + O_2_ (7)

H_2_O_2_ + hν → 2OH (8)

MB + Reactive species (h^+^, e^−^, ·OH, ·OOH or O_2_·^−^) → Intermediates (9)

Intermediates → Degradation products + CO_2_ + H_2_O (10)

### 3.4. The Possible Photocatalytic Mechanism of NO_x_

The mechanism of NO_x_ reduction using pristine and TiO_2_–CaCO_3_ composite catalysts reduces the NO_x_ under UV light irradiation. With the exposure to UV light, the catalyst composite becomes activated, resulting in the generation of electron–hole pairs, which is a crucial step in the photocatalytic process. The composite material remains intact and can continually serve as a photocatalyst, facilitating the reduction of NO_x_ and the oxidation of organic contaminants under UV light exposure. Once the NO_x_ molecules are reduced and organic contaminants are oxidized, they may desorb from the composite surface as harmless gases or byproducts [31]. This process continues as long as there is an energy source, such as UV light, to activate the TiO_2_–CaCO_3_ component of the composite. It offers an eco-friendly approach to reduce NO_x_ emissions and potentially eliminate organic pollutants from the surrounding environment, making it a valuable method for enhancing air quality and reducing the environmental impact of construction materials.

The chemical reactions involved are as follows:

Adsorption of NO_x_:

NO + TiO_2_-CaCO_3_ → NO/TiO_2_-CaCO_3_ (11)

Generation of electron–hole pairs under UV light:

TiO_2_-CaCO_3_ + UV Light (hν) → e^−^ + h^+^ (12)

Reduction of NO_x_ under UV light:

NO/TiO_2_-CaCO_3_ + e^−^ → N_2_ + O_2_ + TiO_2_-CaCO_3_ (13)

These equations illustrate the essential steps in the photocatalytic NO_x_ reduction process using a TiO_2_–CaCO_3_ composite with UV light as the energy source [32,33].

## 4. Results and Discussion

The photocatalytic efficiency of the prepared CaCO_3_–n-TiO_2_ composites, catalysts with various weight percentages (wt.%) of C and T (CT-1, CT-2, CT-3, CT-4, and CT-5), was analyzed. The photocatalytic experiments were carried out in both aqueous as well as in gaseous conditions. Typically, the photodegradation experiments of MB were carried out in aqueous solution under UV irradiation. Gaseous NO_x_ photodegradations were performed with the prepared composite photocatalysts under UV irradiation.

### 4.1. Photodegradation of MB

**Figure 2:**
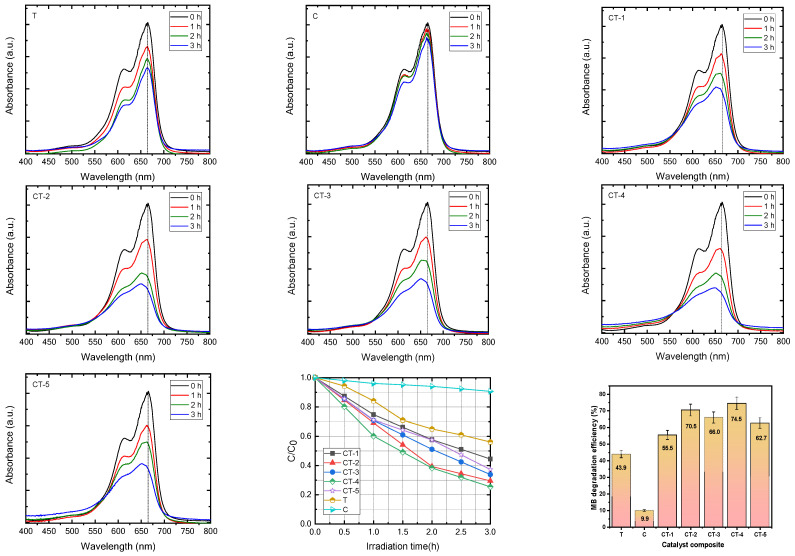
Methylene blue dye degradation and dye degradation efficiency with error bars of C, T, CT-1, CT-2, CT-3, CT-4, and CT-5 composite catalysts.

The photocatalytic activity of the composite catalysts C, T, CT-1, CT-2, CT-3, CT-4, and CT-5 under UV light irradiation was analyzed. The absorbance changes at 665 nm were monitored over the reaction interval of 15 min up to 3 h during the photocatalytic degradation process and are shown in Figure 2. The percentage of MB dye degradation efficiency was calculated with the following equation:

(14) $$\mathrm{Degradation}\;\mathrm{efficiency}\;(\%)=\;\frac{C_{0}-C}{C_{0}}\times 100$$

where C_0_ is the initial concentration of the MB dye solution, and C is the final concentration of the MB dye absorption after irradiation [34].

The photocatalytic degradation of MB by pure T was found to be the slowest as evidenced by the lowest MB-PDE of 44.39% after 3 h (shown in Table 1).

The MB-PDE of C after 3 h is deplorably low (9.40%). The MB-PDE values of the CaCO_3_–n-TiO_2_ composite photocatalysts take the order CT-4 > CT-2 > CT-3 > CT-5 > CT-1 > T > C; the degradation efficiency is shown in Figure 2. The trend indicates that there is variation in the MB-PDE values amongst the composite photocatalysts. The CT-4, at 74.5%, exhibits the highest MB-PDE value. It gives a clue that not only the simple synergistic effect of the two components in the composites but also the composition play an important role in deciding the photocatalytic efficiency of the prepared photocatalysts [35,36,37,38]. The photodegradation of MB under UV irradiation follows the pseudo-first-order kinetics, and the rate constants derived from the slopes of the linear plots are presented in Figure S1. In a comparison of the pristine samples of C and T, the composite samples of CT-1, CT-2, CT-3, CT-4, and CT-5 had increased rate constants in comparison to their pristine counterparts. Consequently, this indicates that there can be a hybrid effect that may influence the rate of MB photodegradation [39,40]. The rate constant comparison informs that the CT-4 samples showed the highest photodegradation rate constants for MB photodegradation amongst the photocatalysts tested. The rate constant for the CT-4 samples is typically reported to be 0.45 s^−1^, which is considerably higher than the rate constant for pure T samples (0.2 s^−1^) as well as the rate constant for C samples (0.03 s^−1^), which indicates that there has been a hybrid effect between the C and T photocatalysts on the composite CT-4 photocatalytic properties [41].

### 4.2. Photodegradation of NO_x_

**Figure 3:**
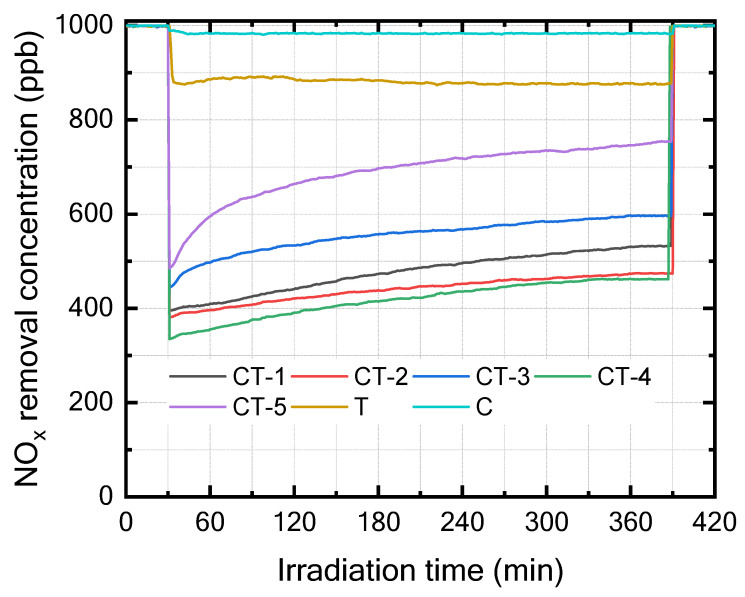
NO_x_ removal of T, C, CT-1, CT-2, CT-3, CT-4, and CT-5 composite catalysts.

The photocatalytic performance of pristine and CaCO_3_–n-TiO_2_ composite catalysts with varying weight percentages of CaCO_3_ and n-TiO_2_ (C and T) was evaluated for NO_x_ removal under UV light at room temperature and is shown in Figure 3. The catalyst exhibited effective photocatalytic activity, leading to a notable decrease in the concentrations of NO_x_. The NO_x_ removal performance was computed using the following equation:

(15) $${NO}_{x}\;removel\;efficiency\left(\%\right)=\frac{{NOx}_{in}-{NOx}_{out}}{{NOx}_{in}}\times 100$$

where NO_x_ represents the initial concentration of NO_x_, and NO_x out_ is the recorded concentration at the end of the photodegradation process [32].

**Figure 4:**
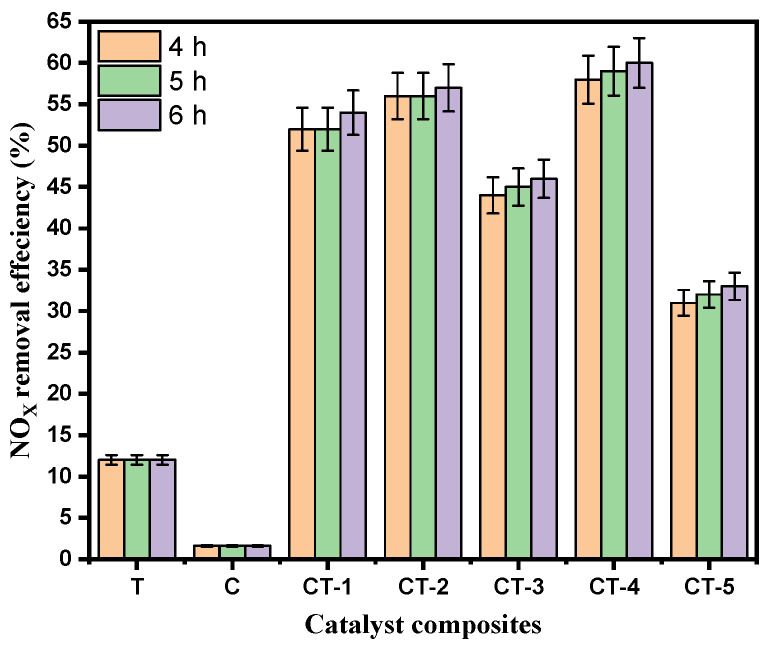
Photocatalytic NO_x_ degradation efficiency with error bars of T, C, CT-1, CT-2, CT-3, CT-4, and CT-5 composites.

Figure 4 presents the bar chart indicating the comparison of the NO_x_ removal efficiency (%) of the various composites and pure components (C and T). The results in Figure 3 and Figure 4 show that CT-4 exhibited excellent NO_x_ photodegradation compared with the other composites and pure components.

Figure 4 shows the pristine and different concentrations of the composite samples of NO_x_ degradation efficiency between the mortar, pristine, and composite photocatalysts. The mortar sample that showed the lowest NO_x_ photodegradation efficiency of 1.6% was the sample mixed with the C sample. In contrast, the CT-4 composite samples exhibited the highest NO_x_ photodegradation efficiency of 60% during 6 h. The observed trends in photocatalytic NO_x_ removal efficiencies suggest a complex interplay between CaCO_3_ and TiO_2_ content in the CaCO_3_–n-TiO_2_ composite catalysts. The catalyst CT-4 highlights the importance of an optimal balance between CaCO_3_ and n-TiO_2_ for the highest NO_x_ removal. This removal efficiency can be attributed to the synergistic effect between CaCO_3_ and n-TiO_2_, where the higher percentage of n-TiO_2_ appeared to enhance the catalytic activity for NO_x_ degradation. The increase in n-TiO_2_ content seemed to have a marginal effect on the catalyst performance, suggesting a potential threshold or optimal balance between carbon and titanium dioxide content for enhanced photocatalytic activity.

### 4.3. NO_x_ Photodegradation of Catalyst CT-4 Mixed Cement Mortar

The highest efficiency NO_x_ removal catalyst CT-4 mixed cement mortar possesses excellent photocatalytic activity amongst the composites; studies were performed to know the influence of coating of CT-4 over the mortar surface on the photocatalytic NO_x_ removal efficiency. This study aimed to develop environment-friendly construction materials for photocatalytic NO_x_ degradation in cement mortar, and the results are shown in Figures S2 and S3. The photocatalyst CT-4 was mixed with cement mortar at various ratios (0%, 5%, 10%, and 15%) as shown in Table 2.

**Figure 5:**
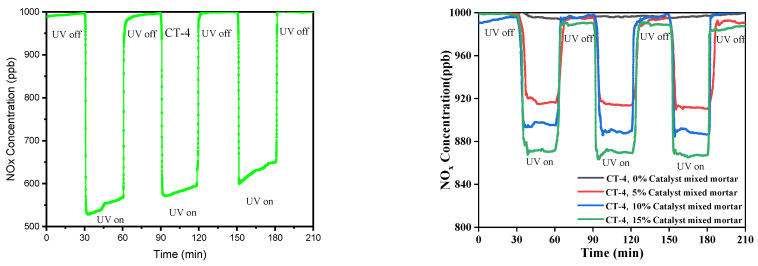
The CT-4 catalyst composite surface coated on mortar NO_x_ removal performance of UV on–off cycles and photocatalytic CT-4 composite 0%, 5%, 10%, 15% mixed cement mortar for UV on–off cycles.

To evaluate the effectiveness of NO_x_ photodegradation, plain mortar (0%) was used as a reference sample, and the average NO_x_ concentrations were analyzed during three segments of NO_x_ degradation as 30 min off state, followed by 30 min on state, and this cycle was performed three times during the 210 min as shown in Figure 5.

The NO_x_ degradation was measured for the mortar samples mixed with the CT-4 photocatalyst at different inclusion levels. The results indicated that the NO_x_ removal increased with an increase in the weight percentage of the photocatalyst on the surface. The average NO_x_ concentrations for the mortar mixed with the CT-4 catalyst at 5%, 10%, and 15% inclusions were 895 ppb, 866 ppb, and 825 ppb, respectively. Moreover, the mortar mixed with the CT-4 photocatalyst at 15% exhibited higher NO_x_ photodegradation efficiency than the mortar mixed with 5% and 10%. This could be attributed to the larger component amount of CT-4 catalyst encompassed on the mortar surface during the manufacturing process. It can be observed that the mortar coated with 15% photocatalyst had the highest NO_x_ degradation efficiency, indicating the potential effectiveness of a reduced content of coating of CT-4 catalyst and suggesting that CT-4 can be affected and used as the economical additive with mortar for NO_x_ removal in cement mortar.

### 4.4. UV–Visible Analysis

**Figure 6:**
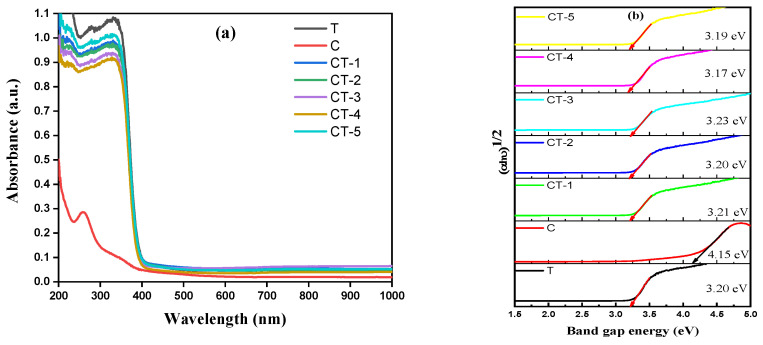
(**a**) UV–DRS and (**b**) Tauc plots of T, C, CT-1, CT-2, CT-3, CT-4 and CT-5 composites.

The UV–DRS spectra of the pristine and CaCO_3_–n-TiO_2_ composite exhibited characteristic absorption features in the ultraviolet to visible range and are shown in Figure 6a.

A wide absorption band in the range between 250 nm and 400 nm was observed. The spectra demonstrated distinctive absorption peaks and shoulder regions, indicative of the electronic transitions within the materials. The band gap energy (Eg) of the catalyst composite was determined from the following relation:

αhν = A(hν − Eg)^n/2^ (16)

where α is the optical absorption coefficient, hν is the photon energy, Eg is the energy band gap, A is the constant, and n = 1 and n = 2 are the direct and indirect band gap semiconductors [34]. The optical energy transition of the CaCO_3_–n-TiO_2_ composite is directly transition allowed, so n = 1. the spectra analysis revealed distinct absorption features for each composition, while the band gap analysis provided valuable insights into the optical properties of the CaCO_3_–n-TiO_2_ composite catalysts with varying C and T weight percentages. The plot of (αhν)2 vs. hν catalyst composite is shown in Figure 6b, which depicts the variation energy band gap concerning composite compositions. The energy band gap of the n-TiO_2_ indirect transition was calculated as 3.20 eV, and that of CaCO_3_ was calculated as 4.15 eV. The composites of CaCO_3_–n-TiO_2_ with various compositions of CT-1, CT-2, CT-3, CT-4, and CT-5 band gap values are 3.21, 3.20, 3.23, 3.17, and 3.19 Ev for the composite catalysts. It shows that the CaCO_3_-n-TiO_2_ composite exhibits similar light absorption of n-TiO_2_. This result shows that the similar properties of n-TiO_2_ consist of strong photolytic degradation properties [42,43]. These findings lay a foundation for further understanding the photocatalytic activities and potential applications of these composite catalyst materials.

### 4.5. Micro-Structure Analysis

**Figure 7:**
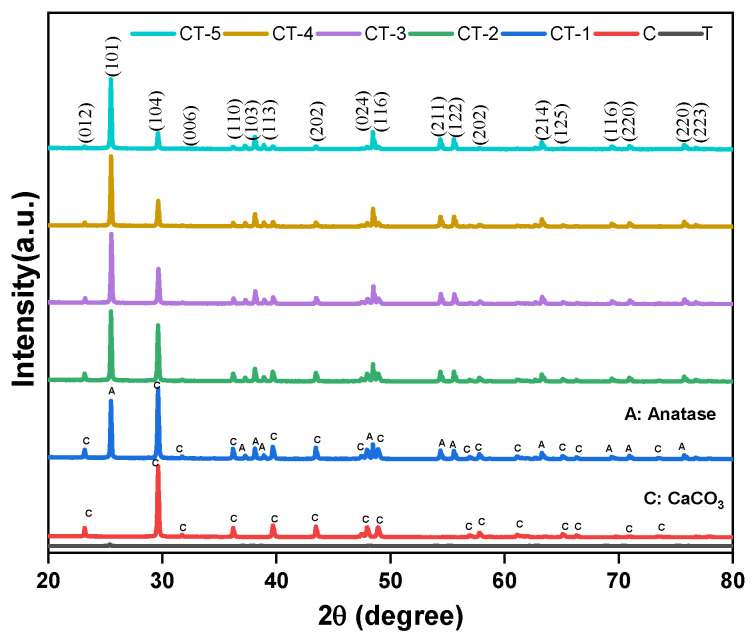
Powder XRD pattern of T, C, CT-1, CT-2, CT-3, CT-4, and CT-5 composites.

The powder X-ray diffraction patterns of the CaCO_3_–n-TiO_2_ composites of various compositions (CT-1, CT-2, CT-3, CT-4, and CT-5) are presented in Figure 7. For comparison purposes, the XRD patterns of pure T and C are also displayed. The diffraction peaks are observed at 25.4°, 37.8°, 48.1°, and 54.0° for T and CaCO_3_–n-TiO_2_ composites that are assigned to the (101), (004), (200), and (211) crystal planes of the anatase phase (JCPDS No. 00-071-1166 and JCPDS No. 00-083-0578). The peak shape and position of the CaCO_3_–n-TiO_2_ composites are approximately the same as those of pure anatase TiO_2_ (T). However, the intensities of the anatase peaks are suppressed. Keeping this information, it is envisaged that TiO_2_ exits on the surface of CaCO_3_. The sharp and narrow peaks of CaCO_3_ are assigned to the diffraction planes (hkl) planes (012), (104), (110), (113), (202), (018), (116), and (122) crystalizing planes of calcite phase of CaCO_3_ [44]. There is an increased intensity of characteristic diffraction peaks belonging to C in conjunction with the decreased intensity of TiO_2_ diffraction in TiO_2_–CaCO_3_ composite samples following the influence of TiO_2_ on the surface of CaCO_3_ [43].

The coexistence of diffraction peaks of the calcite and anatase phases in the CaCO_3_–n-TiO_2_ composites suggests that the crystalline nature of the pure components is retained in the composites. (Table 3). The crystalline unit cell parameters of T (tetragonal) and C (trigonal) are derived from the major diffraction (101) and (104) peaks of T and C, respectively (Table 3). On perusal of Table 3, it can be inferred that the lattice constants a and c of the anatase phase are increased from 3.752 Å and 9.405 Å to a higher value.

On the contrary, the lattice constant a of C remains unaltered upon composite formation, and the value of c decreased from pure C (c = 17.050 Å) to a lesser value. This information suggests that, during composite formation, the c-axis of T (anatase phase) is elongated, possibly by the insertion of Ca ions. The average crystallite size was obtained using the Scherrer formula:

(17) $$D=\frac{K\lambda }{\beta \cos\theta }$$

where D is the crystalline size, λ is the wavelength of Cu–kα radiation (1.54056 Å), β is the full-width half maximum of peak intensity, and θ is the peak position. The crystallite sizes of T and C were calculated from the (101) and (104) diffraction peaks of T and C, respectively. The crystallite size of the composites calculated based on (101) diffraction peak of T (T-50.31 nm, CT1-53.83, CT2-54.02, CT3-54.83, CT4-54.4 and CT5-58.12) was found to be slightly larger than the size of pristine T. Based on that, it can be inferred that there can be a surface layer of C on the surface of the T. And the size of the composites calculated based on (104) diffraction peak of C showed a decrease as compared to pristine C (Table 3) and an increase as compared to pristine T. The coating of C over T or T over C could be the reason for the changes in the sizes of the composite particles.

**Figure 8:**
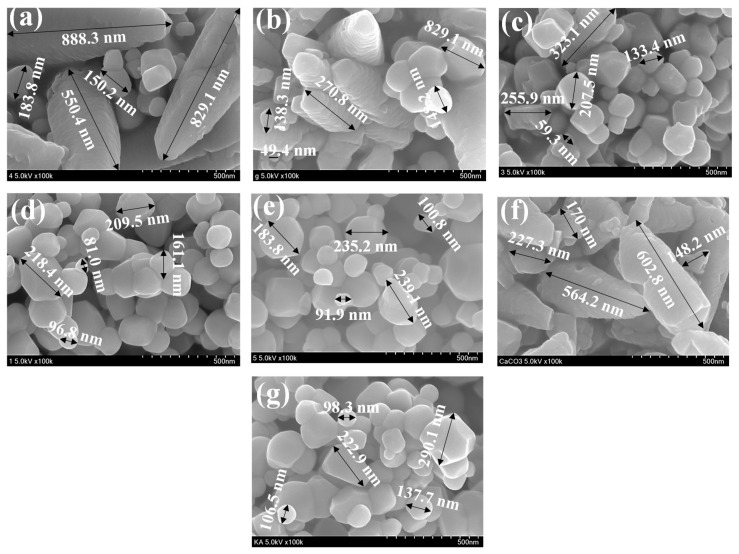
Microstructural images of (**a**) CT-1, (**b**) CT-2, (**c**) CT-3, (**d**) CT-4, (**e**) CT-5, (**f**) C, and (**g**) T composites.

The morphology of the T, C, CT-1, CT-2, CT-3, CT-4, and CT-5 composites are shown in Figure 8a–g.

The existence of irregularly shaped granular TiO_2_ particles of different sizes (with particle sizes of 0.05–0.60 µm) can be observed with good dispersity. We consider that smaller particles are agglomerated to be present as larger-sized particles [45]. Figure 8g presents the morphology of pure C. It can be observed that C existed rhombohedral phased calcite with a cubical morphology as a major component and hexagonal phased vaterite with spherical morphology. Figure 8a–e present the morphologies of the various composites (CT-1, CT-2, CT-3, CT-4, and CT-5), which suggest that particles with mixed morphologies of both C and T and the sizes of the particles are dependent on the composition of C and T in the composites. On close analysis of Figure 8a–e, the average particle size is higher for CT-1, CT-2, and CT-3, the composites having larger proportions of C in the composite. The average particle sizes of CT-4 and CT-5 (the composites having higher proportions of T) are comparatively smaller. Keeping the morphological and size variations amongst the composites, we envisage coating of C over T for CT-1, CT-2, and CT-3 and coating of T over the surface of C for CT-4 and CT-5. The observed higher photocatalytic efficiency for CT-4, as noticed in Figure 8a–c, can be due to the existence of more T phases on the surface of C and the hybrid effect of C and T on the photocatalytic activity.

### 4.6. Elemental and Electronic States

**Figure 9:**
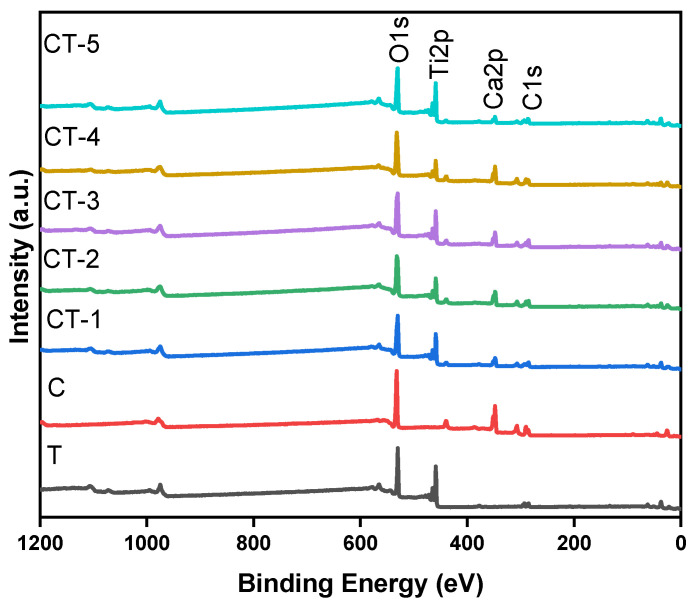
XPS survey spectrum of CT-1, CT-2, CT-3, CT-4, and CT-5 composites and pristine components (C and T).

XPS was used for the qualitative surface elemental composition and quantitative states of the various elements. Figure 9 shows the XPS survey spectra for the TiO_2_–CaCO_3_ composites (CT-1, CT-2, CT-3, CT-4, and CT-5) and their precursors.

**Figure 10:**
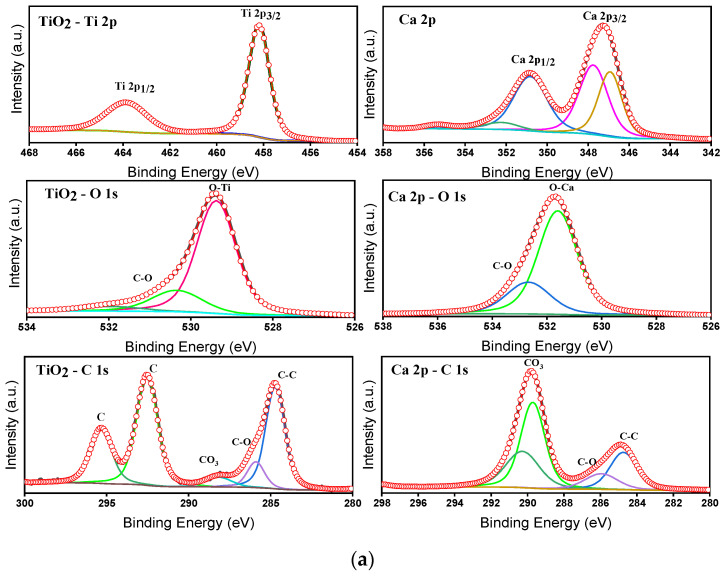

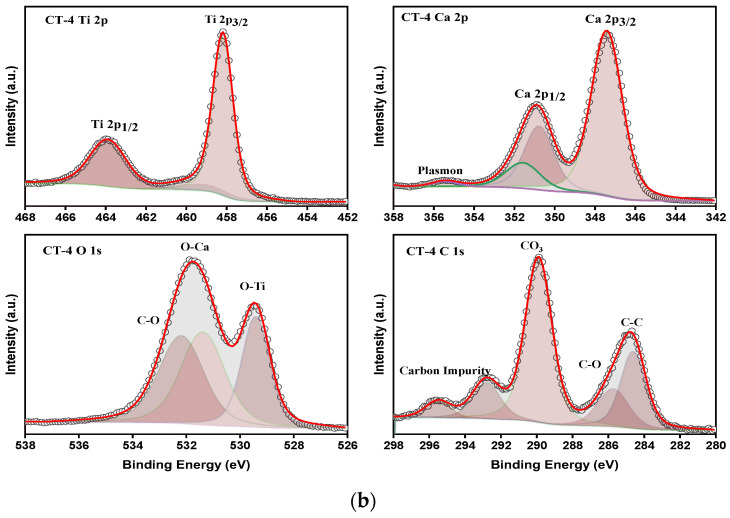
(**a**) Deconvoluted XPS core level spectra of Ti2p, Ca2p, O1s, and C1s peaks for the T and C composites. (**b**) Deconvoluted XPS core level spectra of Ti2p, Ca2p, O1s, and C1s peaks for the CT-4 composites.

The survey spectra inform the existence of Ca, Ti, and O as major elements through the respective binding energy peaks. The absence of other elements indicates the purity of the prepared composite samples. The individual core level spectra for the elements T, C, CT-1, CT-2, CT-3, CT-4, and CT-5 are shown in Figure S4. The deconvoluted individual core level spectra for the elements T and the CT-4 composite mixture were depicted in Figure 10a,b.

The charge correction for the core level peak positions was carried out by C1s (284.8 eV) as a reference. The core level spectrum of Ti2p of T exhibits binding energy (BE) peaks at 458.2 and 464.9 eV, corresponding to the spin–orbit splitting of 458.2 and 464.9 eV relevant to Ti2p3/2 and Ti2p1/2 electronic states with a BE difference of 6.70 eV.

This indicates the presence of Ti^4+^ valance states [28]. Further, the BE peak position of Ti2p is shifted towards higher energy for CT-4, informing the probable changes in the electronic state of Ti^4+^ (Figure 10b). The result is consistent with the XRD results (Table 3), informing the changes in the lattice constants tetragonal phase of TiO_2_. Considering the ionic radius of Ti^4+^ and Ca^2+^ is 0.064 nm and that of Ti^4+^ is 0.106 nm, doping of TiO_2_ by Ca ions is expected [46]. Figure 10a,b compare the BE peaks of Ca 2p. The BE peaks are noticed for pure C at 347.2 eV and 350.9 eV, corresponding to Ca 2p1/2 and Ca 2p3/2, which are in good agreement with the Ca^2+^ oxidation state, respectively [47]. Furthermore, there was a small shift observed in the binding energy peaks of Ca 2p for CT-4 (Figure 10b). The absence of satellite peaks and individual core level peak splitting in the composite samples details the elemental and phase purity of the samples. The core level spectra of O1s are approximately 530 eV in all pure and composite samples. Moreover, the case of pure samples (TiO_2_ and CaCO_3_) shows a maximum of 529.5 eV and approximately 531.6 eV. These binding energy maximums correspond to the metal–oxygen and carbon–oxygen bonding in the TiO_2_ and CaCO_3_ structures. In the case of the composite samples, these two O1s core energy peak maximums are retained without appreciable changes. The deconvoluted peaks at 529.5 eV and 531.6 eV in the composite samples describe the success of composite formation on the catalytic products [48]. The C1s core level spectrum of CT-4 shows BE peaks at 284.8 eV and 290 eV, informing the bonding in the CaCO_3_ structure. From the above observations, we could conclude that CT-4, having changed in the electronic states of Ti^4+^ and Ca^2+^ due to possible interaction during preparation, could cause enhanced photocatalytic effects as compared to pure T.

## 5. Conclusions

In this study, we developed a facile and cost-effective method for the large-scale production of photocatalysts using CaCO_3_-loaded n-TiO_2_ cementitious composites. The optimal balance between CaCO_3_ and n-TiO_2_ influences the catalyst efficiency for degrading MB dye solution and NO_x_ under UV light exposure. Under the influence of UV light irradiation, the high-energy photons activate to create photoinduced electrons (e^−^) and holes (h^+^) in TiO_2_. As the TiO_2_–CaCO_3_ catalyst composite is activated with UV light, electron–hole pairs are generated, making a crucial phase in the photocatalytic process. Especially, the higher ratio of n-TiO_2_ exhibits heightened catalytic activity, suggesting a synergistic relationship between the two constituents characterized with physiochemical analyses. The observed trends in the photodegradation of NOx underscore a nuanced interplay between the CaCO_3_ and TiO_2_ content within the CaCO_3_–n-TiO_2_ composite catalysts. This efficient pollutant removal is attributed to a synergistic effect between CaCO_3_ and n-TiO_2_, where a higher percentage of n-TiO_2_ notably enhances the photocatalytic activity. The CT-4 photocatalyst exhibits exceptional degradation performance, making it a promising application for environmentally friendly and cost-effective construction materials.

## Figures and Tables

**Table 1:** Methylene blue photodegradation of T, C, CT-1, CT-2, CT-3, CT-4, and CT-5 composites.

Irradiation Time (h)	Photocatalysts						
	C	T	CT-1	CT-2	CT-3	CT-4	CT-5
	The Concentration of the Photodegraded Methylene Blue (mg/L)						
1 h	9.98	8.41	7.47	6.91	7.08	6.02	7.11
2 h	9.40	6.5	5.77	3.94	5.11	3.83	5.75
3 h	9.06	5.61	4.45	2.95	3.39	2.55	3.73

**Table 2:** Preparation of mortar and mortar mix with photocatalyst proportion.

Compositions	Cement (g)	Sand (g)	Photocatalysis (wt.%)	Water (%)
Mortar	1	2.5	0	60
Mix catalyst	1	2.5	5	60
Mix catalyst	1	2.5	10	60
Mix catalyst	1	2.5	15	60

**Table 3:** The powder XRD parameters of T, C, CT-1, CT-2, CT-3, CT-4, and CT-5 composite catalysts.

Samples	Composition		2θ (Degree)		The Crystallite Size (nm)		D-Spacing(Å)		Lattice Constants (a, b, c)			
	A (%)	C (%)	A	C	A	C	A	C	A		C	
			(101)	(104)					a = b	c	a = b	c
T	100	-	25.385	-	50.31	-	3.510	-	3.752	9.405	-	-
C	-	100	-	29.379	-	52.65	-	3.037	-	-	4.999	17.050
CT-1	25.1	74.9	25.316	29.400	55.97	51.70	3.515	3.035	3.783	9.508	4.997	17.034
CT-2	34.0	66.0	25.313	29.399	56.34	51.70	3.515	3.035	3.783	9.508	4.997	17.038
CT-3	43.9	56.1	25.301	29.388	58.27	51.39	3.517	3.036	3.785	9.514	4.999	17.042
CT-4	53.5	46.5	25.317	29.401	57.10	51.70	3.515	3.035	3.783	9.509	4.996	17.036
CT-5	66.2	33.8	25.325	29.420	57.87	58.38	3.513	3.033	3.781	9.510	4.993	17.025

## Data Availability

Data and/or samples of the compounds are available from the authors.

## Supplementary Materials

The following supporting information can be downloaded at: https://www.mdpi.com/article/10.3390/nano14020136/s1. Figure S1. Cement mortars mixed with 5%,10%, 15% catalyst composite. Figure S2. Pseudo-first order kinetic profile for photocatalytic degradation of M.B relation between loga-rithm concentration of M.B (C) and time of T, C, CT-1, CT-2, CT-3, CT-4, CT-5 composites. Figure S3. CT-4 composite catalyst painted on the surface of cement mortar. Figure S4. XPS spectrum of (**a**) Ti2P, (**b**) C1s, (**c**) Ca2p, (**d**) O1s of T, C, CT-1, CT-2, CT-3, CT-4, CT-5 catalyst composites.
