# Supplementary material for: Development of TiO2–CaCO3 Based Composites as an Affordable Building Material for the Photocatalytic Abatement of Hazardous NOx from the Environment
Source: Nanomaterials (Basel). 2024 Jan 6;14(2):136. doi: 10.3390/nano14020136 (PMC10819092; doi:10.3390/nano14020136)
Supplement: Supplementary file 1 [file nanomaterials-14-00136-s001.zip › nanomaterials-2789993-supplementary.pdf]

nanomaterials

Supplementary Materials

Development of TiO_2–$CaCO_3$ Based Composites as an Affordable Building Material for the Photocatalytic Abatement of Hazardous NO_x from the Environment

This study aimed to develop environment-friendly construction materials for photocatalytic NOx degradation in cement mortar mixed with 5%,10%, 15% catalyst composite are shown in Fig.S1.

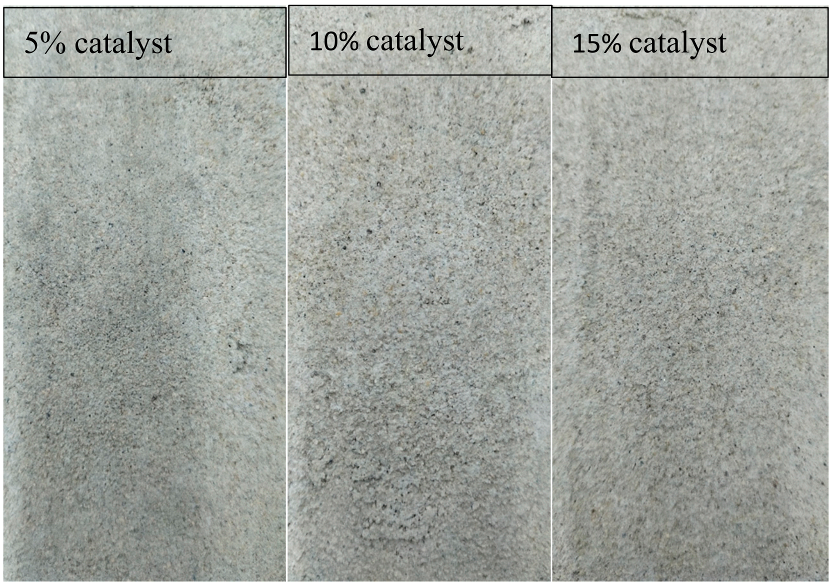

Figure.S1. Cement mortars mixed with 5%,10%, 15% catalyst composite.

The degradation of MB dye under irradiation pseudo-first-order kinetics shown in Fig.S1.

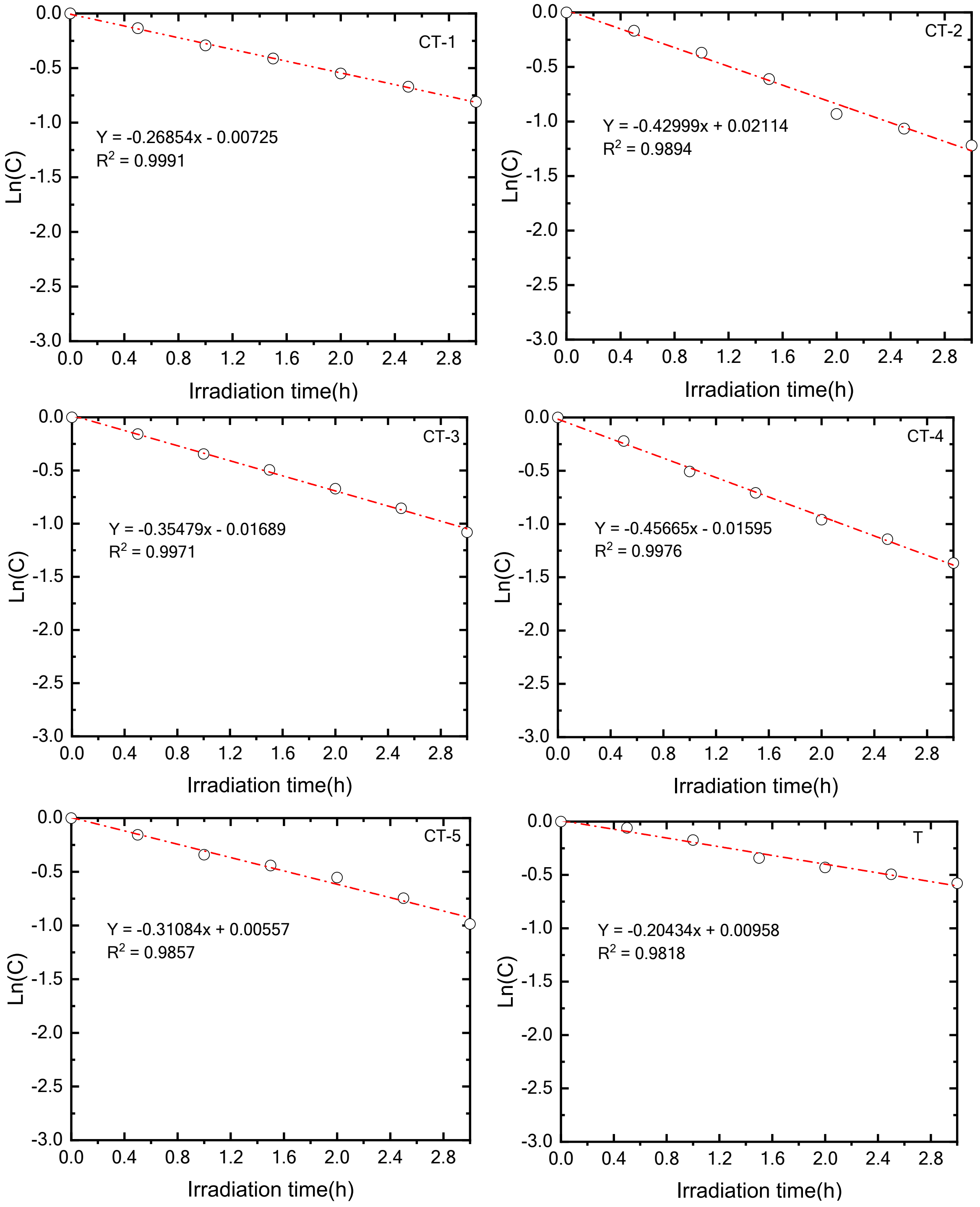
CT-1
Y = -0.26854x - 0.00725
R^2 = 0.9991
CT-2
Y = -0.42999x + 0.02114
R^2 = 0.9894
CT-3
Y = -0.35479x - 0.01689
R^2 = 0.9971
CT-4
Y = -0.45665x - 0.01595
R^2 = 0.9976
CT-5
Y = -0.31084x + 0.00557
R^2 = 0.9857
T
Y = -0.20434x + 0.00958
R^2 = 0.9818
Ln(C)
Irradiation time(h)

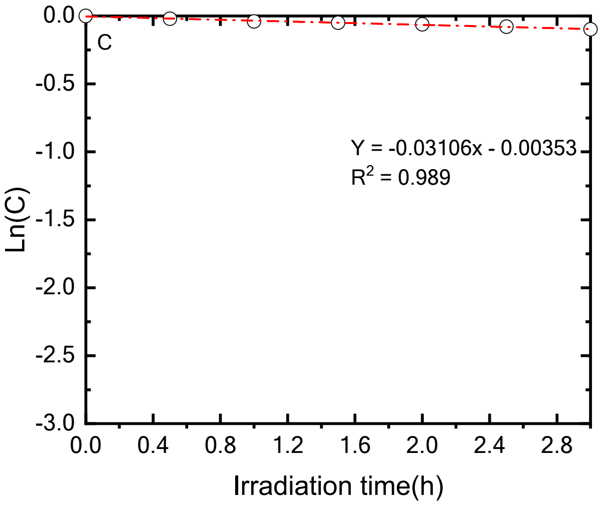

Figure.S2. Pseudo-first order kinetic profile for photocatalytic degradation of M.B relation between logarithm concentration of M.B (C) and time of T, C, CT-1, CT-2, CT-3, CT-4, CT-5 composites.

The silica sol binder with CT-4 catalyst painted on the surface of cement mortar and calcined at 200˚C for 60 minutes as shown in Fig.S3.

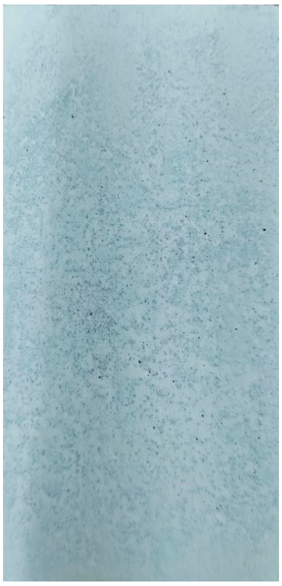

Figure.S3. CT-4 composite catalyst painted on the surface of cement mortar.

The individual core level spectra for the elements in the T, C, CT-1, CT-2, CT-3, CT-4 and CT-5 composite mixture were depicted in Fig.S4.

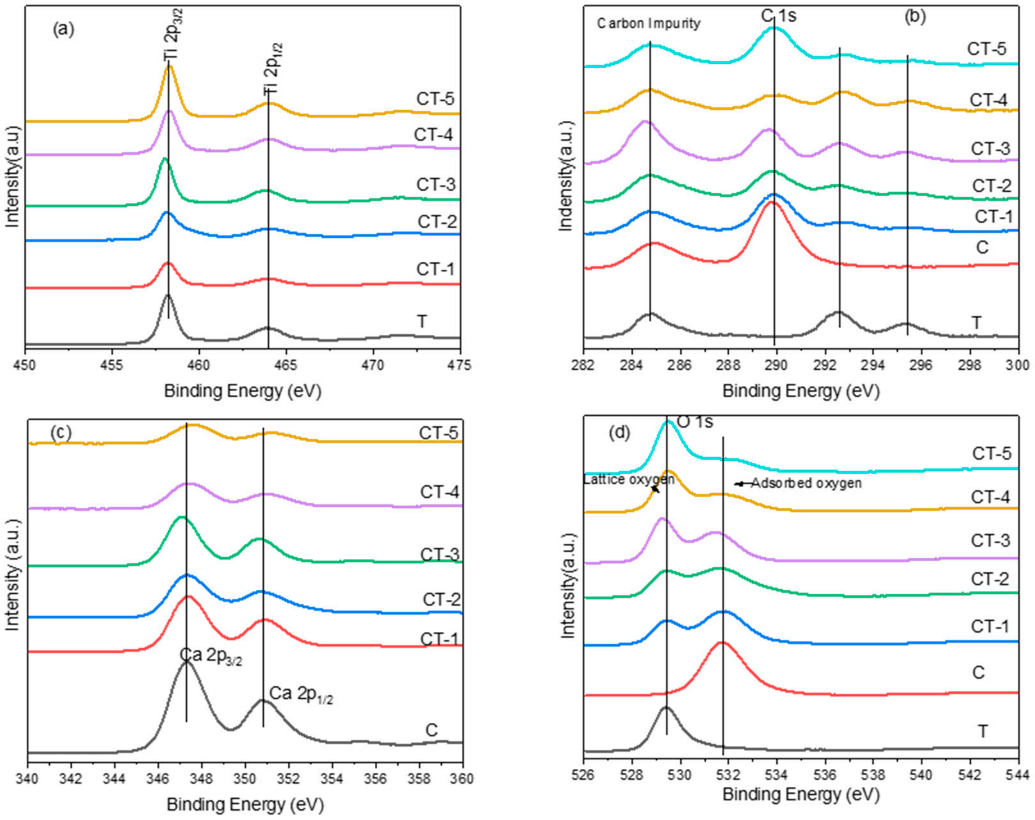

Figure.S4.XPS spectrum of (a) Ti$_{2P}$, (b) C1s, (c) Ca$_{2p}$, (d) O1s of T, C, CT-1, CT-2, CT-3, CT-4, CT-5 catalyst composites.
